# Supplementary material for: Characterization of a Syngeneic Orthotopic Model of Cholangiocarcinoma by [18F]FDG-PET/MRI
Source: Cancers (Basel). 2024 Jul 19;16(14):2591. doi: 10.3390/cancers16142591 (PMC11275149; doi:10.3390/cancers16142591)
Supplement: Supplementary file 1 [file cancers-16-02591-s001.zip › cancers-3103087-supplementary.pdf]

Supplementary Material

Characterization of a syngeneic orthotopic model of cholangiocarcinoma by [^{18}F]FDG-PET/MRI

Lena Zachhuber [1,2], Thomas Filip [3], Behrang Mozayani [4], Mathilde Löbsch [5], Stefan Scheiner [6], Petra Vician [6], Johann Stanek [1], Marcus Hacker [2,7], Thomas Helbich [1,8], Thomas Wanek [1], Walter Berger [6] and Claudia Kuntner [1,7,*]

1 Preclinical Imaging Lab (PIL), Department of Biomedical Imaging and Image-Guided Therapy, Medical University of Vienna, 1090 Vienna, Austria;
2 Division of Nuclear Medicine, Department of Biomedical Imaging and Image-Guided Therapy, Medical University of Vienna, 1090 Vienna, Austria;
3 Institute of Animal Breeding and Genetics & Biomodels Austria, University of Veterinary Medicine, 1210 Vienna, Austria;
4 Department of Pathology, Medical University of Vienna, 1090 Vienna, Austria;
5 Core Facility Laboratory Animal Breeding and Husbandry, Medical University of Vienna, Austria;
6 Centre for Cancer Research and Comprehensive Cancer Center, Division of Applied and Experimental Oncology, Medical University of Vienna, 1090 Vienna, Austria;
7 Medical Imaging Cluster (MIC), Medical University of Vienna, 1090 Vienna, Austria;
8 Division of General and Pediatric Radiology, Department of Biomedical Imaging and Image-Guided Therapy, Medical University of Vienna, 1090 Vienna, Austria;

* Correspondence: claudia.kuntner@meduniwien.ac.at

Table S1. Overview of all animals used in the study.

Summary of animal data						
**Animal Nr.**	**Animal ID**	**Tumor inoculation SB-1**	**Death during/after surgery [ds]**	**Death during imaging [di]**	**Injection failed [F]**	**Technical problems [T]**
1	110	+				
2	111	+				
3	112	+				
4	113	+	ds			
5	114	+				
6	115	+	ds			
7	201	+				
8	202	+		di		
9	203	+		di		
10	204	+				
11	205	+		di		
12	206	+				
13	207	+			F/F*	
14	301	+		di		T
15	302	+			F/F*	
16	303	+	ds			
17	304	+			F/F*	
18	305	+	ds			
19	306	+			F/F*	
20	307	+	ds			
21	120	+				
22	121	+				
23	220	+				
24	221	+	ds			
25	222	+				
26	223	+				
27	224	+				
28	320	+				
29	321	+				
30	322	+	ds			
31	323	+				
32	324	+				
33	325	+		di		
34	326	+				T
35	327	+			F*	T
36	R1	+				
37	K2	-				
38	328	+			F*	T
39	R4	+				
40	R5	+				
41	R6	+				
42	K1	-				
43	K3	-				
44	K4	-				

ds = deaths of animals that occured during surgery (inoculation of tumor cells) or post surgery
di = deaths of animals that occured during the 4 weeks of imaging, during scan time
F = Intraperitoneal injection into the gut
F* = Intraperitoneal injection partially into the gut
T = Technical problems that occur during measurements with imaging devices

Table S2. VOI shapes and sizes used for the quantitative analysis of the dynamic [^{18}F]FDG-PET and [^{18}F]FDG-PET/MR (i. v. injection) images.

Dynamic PET images			
**Organ**	**VOI shape**	**Dimension (mm)**	**Volume (mm³)**
Brain	ellipsoid	7.0 x 5.0 x 10	183.26
Heart	ellipsoid	5.7 x 5.4 x 6.8	109.6
Liver	cube	3.5 x 3.5 x 3.5	43.88
Kidney	ellipsoid	4.2 x 3.4 x 7.0	52.34
Vena cava	cylinder	1.7 x 2.0 x 3.0	7.94

[^{18}F]FDG-PET/MR (i.v. injection) images			
**Organ**	**VOI shape**	**Dimension (mm)**	**Volume (mm³)**
Brain	ellipsoid	7.0 x 5.0 x 10	324.1
Lung	ellipsoid	3.0 x 3.0 x 3.0	14.14
Muscle	ellipsoid	2.4 x 2.4 x 4.0	12.06

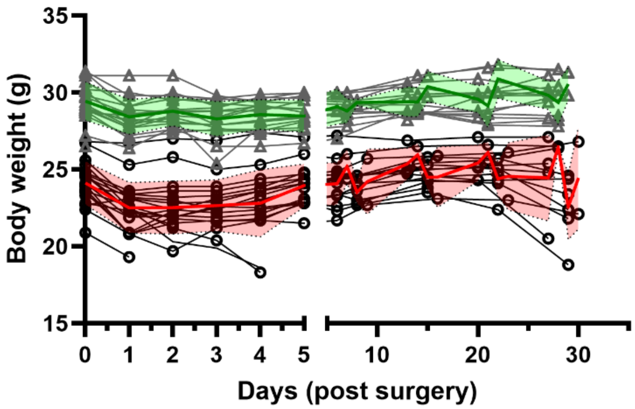

Figure S1. Body weight of the C57BL/6J mice starting with the day of the surgical procedure.

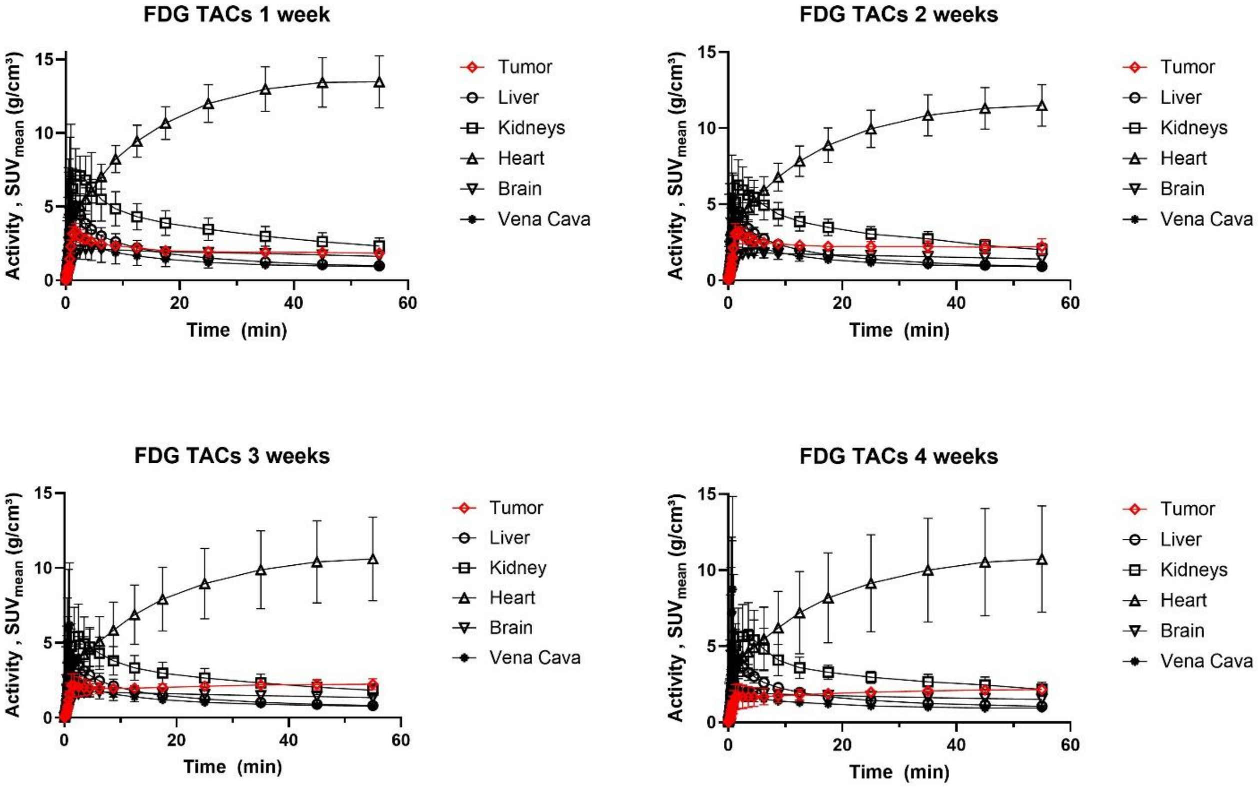

Figure S2. Organ time-activity concentration curves. Expressed as standardized uptake value (SUV) measured after intravenous injection of [^{18}F]FDG in the syngeneic orthotopic CCA model at 1 week, 2 weeks, 3 weeks, and 4 weeks after tumor cell implantation. Data are presented as mean SUV± standard deviation.

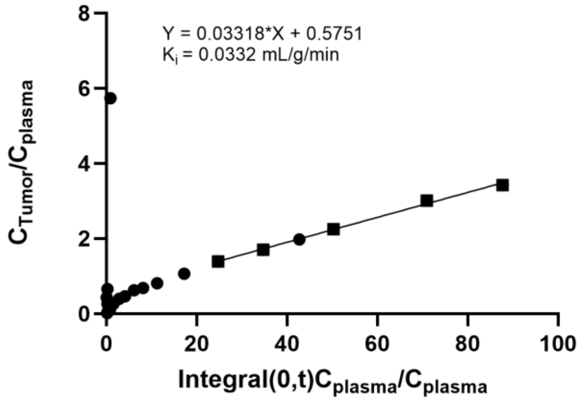

Figure S3. Representative Patlak plot from a tumor extracted from the tumor VOI and image-derived input function. The slope of the linear part of the plot represents the metabolic flux K_i.

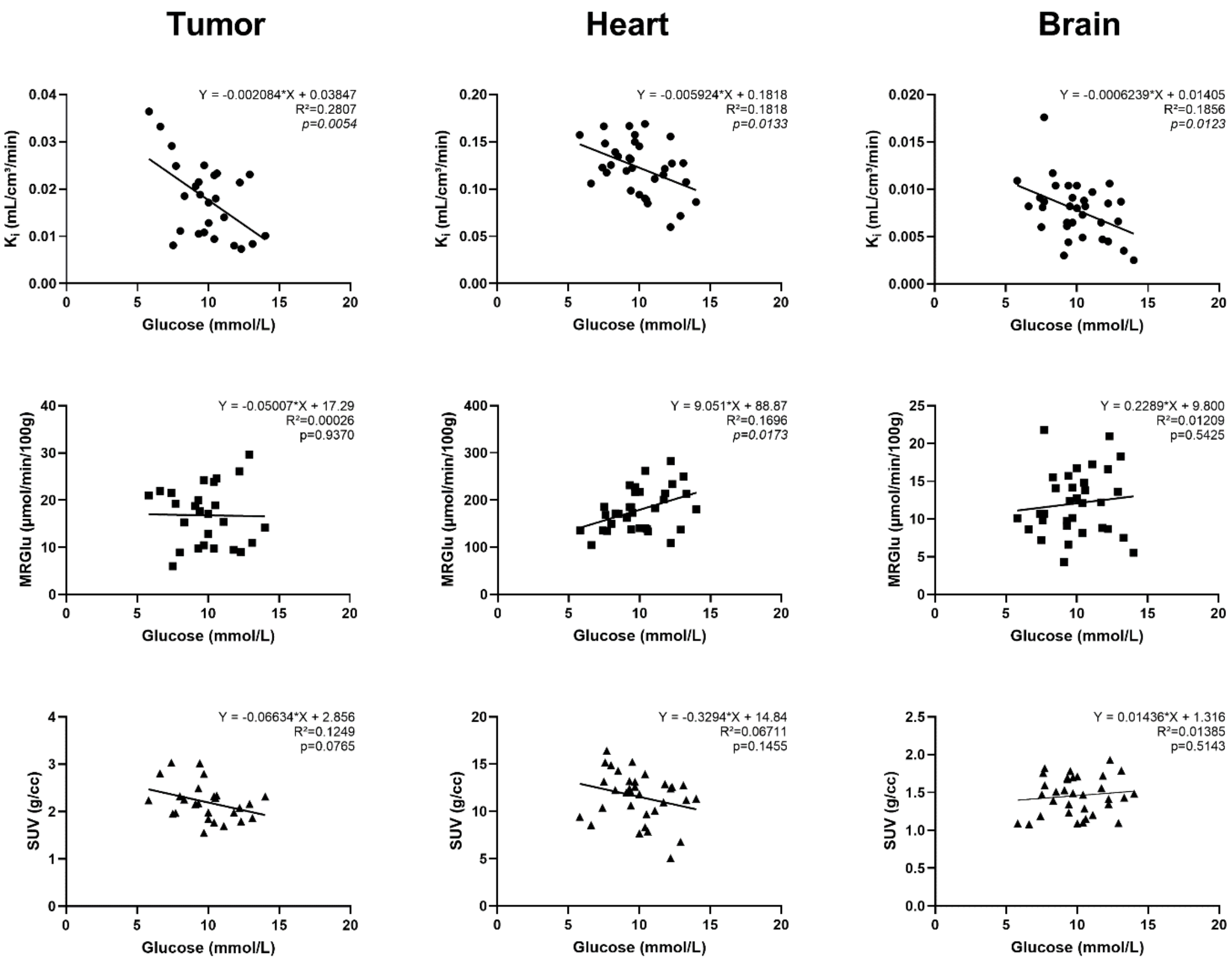

Figure S4. Relationships between blood glucose levels and K_i, *MRGlu*, and *SUV* in tumor, heart, and brain. Pearsons correlation coefficient and the p values are given in the individual plots. For the kursiv written p values is the regression strongly significant at p<0.05.

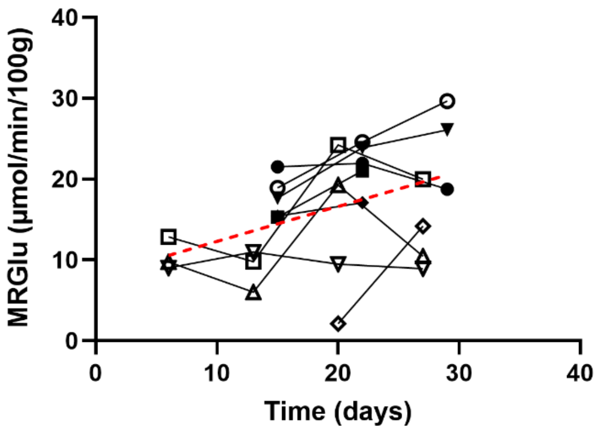

Figure S5. Relationship between *MRGlu* derived from the tumor by Patlak graphical analysis with the time (in days) after tumor cell implantation.

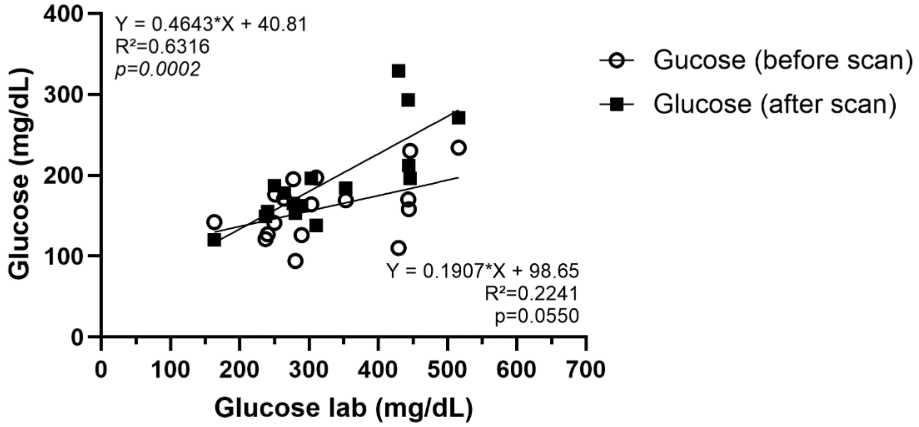

Figure S6. Relationships between glucose levels measured from a certified lab (x-axis; taken from the plasma) and blood glucose levels before and after the PET scan using a commercial glucometer. The measurements taken after the scan are in excellent agreement with the data from the certified lab.
